# Epidemiological and molecular insights into human lice (Anoplura: Pediculidae) in France: genetic diversity and DNA barcoding

**DOI:** 10.1051/parasite/2026060

**Published:** 2026-10-13

**Authors:** Anthony Marteau, Sophie Brun, Arezki Izri, Mohammad Akhoundi

**Affiliations:** 1 Parasitology-Mycology Department, Avicenne Hospital, AP-HP 125, route de Stalingrad 93009 Bobigny cedex France; 2 UMR1137 INSERM, IAME, Sorbonne Paris Nord University France; 3 Unité des Virus Émergents (UVE: Aix-Marseille Univ, Università di Corsica) Marseille France

**Keywords:** Human lice, *Pediculus humanus capitis*, *Pediculus humanus corporis*, *Pthirus pubis*, Genetic diversity, Heterogeneity

## Abstract

Human lice are hematophagous ectoparasites of worldwide distribution and public health concern. Despite their medical importance, the genetic diversity of these ectoparasites remains poorly documented, with only one prior study conducted in France. In this study, we investigated the distribution and genetic diversity of human louse populations collected from patients referred to Avicenne Hospital, northern Paris, between 2019 and 2023. A total of 209 specimens, one per patient, were examined: 186 head lice (*Pediculus humanus capitis*), 11 body lice (*Pediculus h. corporis*), and 12 pubic lice (*Pthirus pubis*). Head lice predominated in women (82.3%), whereas body and pubic lice were more frequent in men (81.8% and 83.3%, respectively). Among head lice patients, 75.8% were children under 18 years, while all body lice patients and 83.3% of pubic lice patients were adults. Two infants with pubic lice were diagnosed, presumably infected by their parents, highlighting transmission risk among family members. Four pubic lice cases involved men who have sex with men. Phylogenetic analysis of cytochrome oxidase I (*COI*) sequences using the maximum likelihood method and comparison to GenBank sequences from multiple regions revealed genetic heterogeneity, with sequences clustering into three monophyletic clades (A, B and C). Clade C is reported for the first time in France. Body louse sequences clustered exclusively within clade A. Together with our previous work, this study represents the first epidemiological investigation of human lice infestation in a general French population, contrasting with earlier reports restricted to schoolchildren. Our findings also improve our understanding of human louse genetic diversity with implications for control strategies.

## Abstract (fr)

Les poux humains sont des ectoparasites hématophages à répartition mondiale représentant un enjeu de santé publique. Malgré leur importance médicale, la diversité génétique de ces ectoparasites reste peu documentée, une seule étude antérieure ayant été menée en France. Cette étude a analysé la répartition et la diversité génétique de populations de poux humains collectés chez des patients pris en charge à l'hôpital Avicenne (au nord de Paris) entre 2019 et 2023. Au total, 209 spécimens (un par patient) ont été examinés : 186 poux de tête (*Pediculus humanus capitis*), 11 poux de corps (*Pediculus h. corporis*) et 12 poux du pubis (*Pthirus pubis*). Les poux de tête prédominaient chez les femmes (82,3 %), tandis que les poux de corps et les poux du pubis étaient plus fréquents chez les hommes (respectivement 81,8 % et 83,3 %). Parmi les patients porteurs de poux de tête, 75,8 % étaient des enfants de moins de 18 ans, alors que tous les patients porteurs de poux de corps et 83,3 % de ceux porteurs de poux du pubis étaient des adultes. Deux nourrissons atteints de poux du pubis ont été diagnostiqués, probablement contaminés par leurs parents, ce qui souligne le risque de transmission au sein de la famille. Quatre cas de poux du pubis concernaient des hommes ayant des rapports sexuels avec des hommes. L'analyse phylogénétique des séquences du gène de la cytochrome oxydase I (*COI*), réalisée par la méthode du maximum de vraisemblance et comparée à des séquences de GenBank provenant de diverses régions, a révélé une hétérogénéité génétique : les séquences se regroupaient en trois clades monophylétiques (A, B et C), le clade C étant signalé pour la première fois en France. Les séquences de poux de corps se regroupaient exclusivement au sein du clade A. En complément de nos travaux antérieurs, cette étude constitue la première enquête épidémiologique sur l'infestation par les poux humains dans une population française générale, contrastant avec les rapports précédents limités aux écoliers, et améliore notre compréhension de la diversité génétique des poux humains, avec des implications pour les stratégies de lutte.

## Introduction

Human lice are obligate hematophagous ectoparasites belonging to the order Phthiraptera and the suborder Anoplura, with a cosmopolitan distribution. Two species of lice parasitize humans: *Pediculus humanus*, which comprises two ecotypes [the head louse (*P. h. capitis*) and the body louse (*P. h. corporis*)], and *Pthirus pubis*, the pubic or crab louse [1, 15].

Head lice (*P. h. capitis*) are obligate human-specific ectoparasites that colonize the scalp and feed exclusively on blood. They are among the most prevalent ectoparasites worldwide, primarily affecting children with a reported prevalence ranging from 0.58 to 67.3%, depending on geographical and socioeconomic factors [13, 17, 22, 39]. Pruritus, which typically occurs on the scalp, retroauricular region, and at the nape of the neck, is the most common symptom caused by head lice. Intense scratching may cause excoriations that predispose to secondary bacterial infections [8, 19]. Despite their ubiquity and the significant discomfort, head lice are not currently recognized as vectors of human pathogens [6].

Body lice (*P. h. corporis*) are other obligate human ectoparasites commonly found in patients’ clothes and are primarily prevalent among homeless individuals and people living in poor hygiene conditions [8, 36]. Clinically, they induce intense pruritus and erythematous papules or wheals at bite sites. Chronic infestations often result in skin thickening, hyperpigmentation, and secondary bacterial infections [11]. Unlike head lice, body lice are proven vectors of several life-threatening pathogens, including *Rickettsia prowazekii* (epidemic typhus), *Bartonella quintana* (trench fever), and *Borrelia recurrentis* (louse-borne relapsing fever) [8, 40]. These diseases have historically caused significant morbidity and mortality, particularly in conditions of overcrowding, poor hygiene, and armed conflict, highlighting the epidemiological importance of body lice as biological vectors.

Pubic lice (*P. pubis*) are parasitic insects that primarily infest pubic hair, but infestations may occasionally involve axillary, thoracic, facial, and, more rarely, scalp hair [2, 15]. Intense pruritus is the most common symptom resulting from a local reaction to the louse’s saliva, sometimes complicated by excoriations and secondary bacterial infections. Transmission occurs mainly through sexual contact. To date, there is no confirmed evidence supporting their role as natural vectors of human pathogens [23].

In recent decades, human lice have been the subject of a number of epidemiological and clinical research studies, increasingly complemented by molecular investigations [1, 30, 32]. Molecular identification and population genetic studies of human louse species have provided crucial insights into their evolution, epidemiology, and vectorial transmission potential. Phylogenetic analyses based primarily on mitochondrial markers such as *Cyt b* and *COI* have demonstrated substantial genetic diversity, leading to the delineation of six major evolutionary clades (A–F) [6, 30, 41].

In France, research on the epidemiology and genetic diversity of human lice remains largely limited. Most studies have focused on head lice in schoolchildren [10, 20, 21] or homeless people [18, 28]. The first phylogenetic study conducted by our parasitology department on the general population identified clades A, B, and E in head lice, whereas body lice were found to belong exclusively to clade A [14]. In continuation of our previous work on the epidemiology and diversity of louse populations in France, particularly in the Île-de-France region, the present study investigated human lice collected from patients referred to a hospital located in the northern suburbs of Paris. Moreover, we evaluated the genetic diversity within various selected louse populations in patients with diverse geographical origins.

## Materials and methods

### Ethics statement

This study was conducted in accordance with the Jardé Law (Law No. 2012-300 of March 5, 2012), Category 3 (RIPH-3), under protocol number 95/99/AVC/ESA, and approved by the Avicenne Hospital Research Ethics Committee. It corresponds to non-interventional research on ectoparasites collected for diagnostic purposes. Informed consent was obtained from the patients (or from the parents of infested children), including authorization for the publication of medical information. All individuals received proper medical care, in addition to the specimen collection for laboratory study.

### Lice collection

Lice specimens were collected from patients referred for diagnosis and treatment of pediculosis at the Parasitology-Mycology Department of Avicenne Hospital (Bobigny, France) between March 2019 and March 2023. Head, body, and pubic lice were gathered using combs or tweezers, placed in Petri dishes, and identified morphologically using an identification key [16, 26]. Epidemiological data (e.g., age, sex, and country of origin or recent travel) and clinical information (e.g., symptoms, treatment history) were then recorded for each patient.

### DNA extraction and PCR amplification

A single adult louse specimen was randomly selected for each patient for molecular analysis. Genomic DNA was extracted using the Chelex 10% method (Bio-Rad, Marnes-la-Coquette, France), following the protocol described by Walsh *et al.* [42]. The extracted DNA was subjected to conventional PCR targeting an 828 bp fragment of the mitochondrial cytochrome oxidase 1 (*cox1*) gene [5]. Each PCR reaction was performed in a final volume of 25 μL, comprising 12.5 μL of master mix (AmpliTaq Gold 360 Master Mix, Applied Biosystems, Foster City, CA, USA), 8 μL of distilled nuclease-free water, 0.5 μL of each of the forward (5′–GGAGTGAGTTCGATTTTAG–3′) and reverse (5′–GTGCTGAGGAAAGAAAGTC–3') primers, and finally 3.5 μL extracted template DNA. Amplification was performed under the following cycling conditions: initial denaturation for 5 min at 94 °C, followed by 40 cycles of 30 s at 94 °C, 1 min at 55 °C, and 1 min at 72 °C, and a final elongation of 5 min at 72 °C. Negative (without DNA template) and positive controls were included in each PCR set to ensure the validity of the amplification process. PCR products were analyzed by electrophoresis on 1.5% agarose gels containing ethidium bromide and visualized under UV illumination.

### Phylogenetic reconstruction and species assignation

PCR amplicons were subjected to bidirectional Sanger sequencing using the same primers employed for amplification. The acquired sequences were edited and aligned, and species-level identification was performed using the Basic Local Alignment Search Tool (BLAST).

Phylogenetic relationships among lice sequences obtained in this study and reference sequences (earliest sequences representing each unique genotype) from GenBank were inferred using the maximum likelihood (ML) method under the Tamura-Nei substitution model. Branch support was assessed by 1,000 bootstrap replicates. The best-fitting nucleotide substitution model was determined using MEGA, version 11 [27], and the Nearest-Neighbor Interchange (NNI) algorithm was employed as the ML heuristic search strategy. In addition to phylogenetic tree reconstruction, population-level analyses were performed to investigate genetic diversity and evolutionary relationships. Haplotype networks were constructed using the median-joining algorithm implemented in PopART, version 1.7. Indices of genetic diversity, including haplotype diversity (Hd), nucleotide diversity (π), and the number of polymorphic sites, were calculated using DnaSP version 6. These analyses provided insights into the genetic structure, demographic history, and geographical distribution of the lice populations studied.

## Results

A total of 209 patients infested with human lice were examined in this study, including 186 cases of head lice, 11 of body lice, and 12 of pubic lice. Head lice were predominantly observed in women (153/186; 82.3%), whereas body and pubic lice were more frequently observed in men (9/11; 81.8% and 10/12; 83.3%, respectively). Furthermore, among patients with head lice, 75.8% (141/186) were children or adolescents under 18 years of age. In contrast, all patients infested with body lice and 83.3% (10/12) of those with pubic lice were adults.

Among patients with body lice, 54.5% (6/11) were homeless, while the remaining patients lived alone in poor hygiene conditions. Among the patients with pubic lice, 33.3% (4/12) were men who have sex with men (MSM), and 16.7% (2/12) were infants presumably infected by their parents. Among the patients with pediculosis, 44.5% were French with no history of travel, while the remaining patients originated from 17 countries across Africa (Algeria, Gabon, Ivory Coast, Mauritius, Morocco, Togo, and Tunisia), Asia (Bangladesh, India, Lebanon, Pakistan, and Sri Lanka), Europe (Italy, Portugal, Romania, and Serbia), and South America (Venezuela). Head lice infestations were most frequent among patients from France (82/186; 44.1%), followed by Sri Lanka (49/186; 26.3%). Body lice were predominantly observed in patients from France (5/11; 45.5%), whereas pubic lice were most common among patients from France (5/12, 41.7%) and Algeria (3/12; 25%). Epidemiological information for the infested individuals and their louse specimens analyzed in this study is provided in Tables 1 and 2, and in Supplementary files SI.I & SI.II.

**Table 1:** Epidemiological features of the patients with pediculosis examined in this study.

Louse species	Patient origin, sex and louse specimen number																																				Total
	Asia										Europe										Africa														South America		
	Sri Lanka		Pakistan		Bangladesh		India		Lebanon		France		Portugal		Serbia		Romania		Italy		Nigeria		Tunisia		Morocco		Gabon		Togo		Mauritius		Ivory Coast		Venezuela		
	♂	♀	♂	♀	♂	♀	♂	♀	♂	♀	♂	♀	♂	♀	♂	♀	♂	♀	♂	♀	♂	♀	♂	♀	♂	♀	♂	♀	♂	♀	♂	♀	♂	♀	♂	♀	
*Pediculus h. capitis*	4	45	1	8	1	7	0	1	0	1	16	66	1	6	0	0	0	0	0	1	5	6	1	9	0	1	0	1	0	0	1	1	3	0	0	0	186
*Pediculus h. corporis*	1	0	1	0	0	0	1	0	0	0	3	2	0	0	1	0	1	0	0	0	0	0	0	0	0	0	0	0	1	0	0	0	0	0	0	0	11
*Pthirus pubis*	0	0	0	0	0	0	0	0	0	0	5	0	1	1	0	0	0	0	0	0	2	1	0	0	1	0	0	0	0	0	0	0	0	0	1	0	12
Total	5	45	2	8	1	7	1	1	0	1	24	8	2	7	1	0	1	0	0	1	7	7	1	9	1	1	0	1	1	0	1	1	3	0	1	0	209

**Table 2:** Age range of the patients with pediculosis examined in this study.

Age category	Human Louse spices			Total
	Head lice	Body lice	Pubic lice	
1–5	20	0	2	22
6–10	66	0	0	66
11–15	44	0	0	44
15–20	12	0	0	12
21–25	4	0	0	4
26–30	8	0	0	8
31–35	6	1	1	8
36–40	7	2	2	11
41–45	9	1	1	11
46–50	5	2	0	7
51–55	4	0	2	6
56–60	0	0	0	0
61–65	0	0	0	0
66–70	0	1	1	2
71–75	1	1	3	5
76–80	0	1	0	1
81–85	0	2	0	2

BLAST analysis of the obtained *COI* sequences revealed more than 97% identity with homologous reference sequences retrieved in GenBank. All nucleotide sequences were deposited in GenBank with the assigned accession numbers of PZ771150–PZ771240 (SI.III). Phylogenetic analysis of *COI* sequences of louse specimens processed in this study along with homologous GenBank sequences using the ML method demonstrated heterogeneity, with the analyzed head louse sequences clustering into three main mitochondrial lineages. While the first clade (clade A) represented the largest and most geographically heterogeneous lineage composed of head louse specimens at the international scale (e.g., Algeria, Bangladesh, France, Ivory Coast, Lebanon, Morocco, Pakistan, Portugal, Sri-Lanka, and Tunisia), the second clade (clade B) included the louse samples from Algeria, France, Portugal, and Tunisia. The third clade (clade C) was comprised of the sequences from Algeria, France, Gabon, India, Mauritius, Pakistan, Sri-Lanka, and Tunisia. In addition, body lice sequences analyzed in this study clustered within clade A, indicating no clear phylogenetic separation according to host ecotype between head and body lice within this clade. This finding suggests that the *COI* marker did not resolve a mitochondrial differentiation corresponding to these two ecotypes (Fig. 1). Furthermore, the clustering observed in the median-joining network corroborated the topology of the phylogenetic trees generated by the ML analysis (Fig. 2). The median-joining network revealed six haplotypes with a haplotype diversity of 0.6568. Three predominant haplotypes (H1–H3) comprised mainly *P. h. capitis* sequences and showed broad geographic distribution, whereas H4 and H5 corresponded to *P. h. corporis*. Some body louse sequences shared H1 and H3 with head lice, indicating that these mitochondrial haplotypes were not strictly host-form specific. *Pediculus schaeffi* formed a distinct outgroup haplotype (H6). Haplotype frequency and estimated genetic distance within louse specimens is provided in Supplementary Information SI.IV.

**Figure 1:**
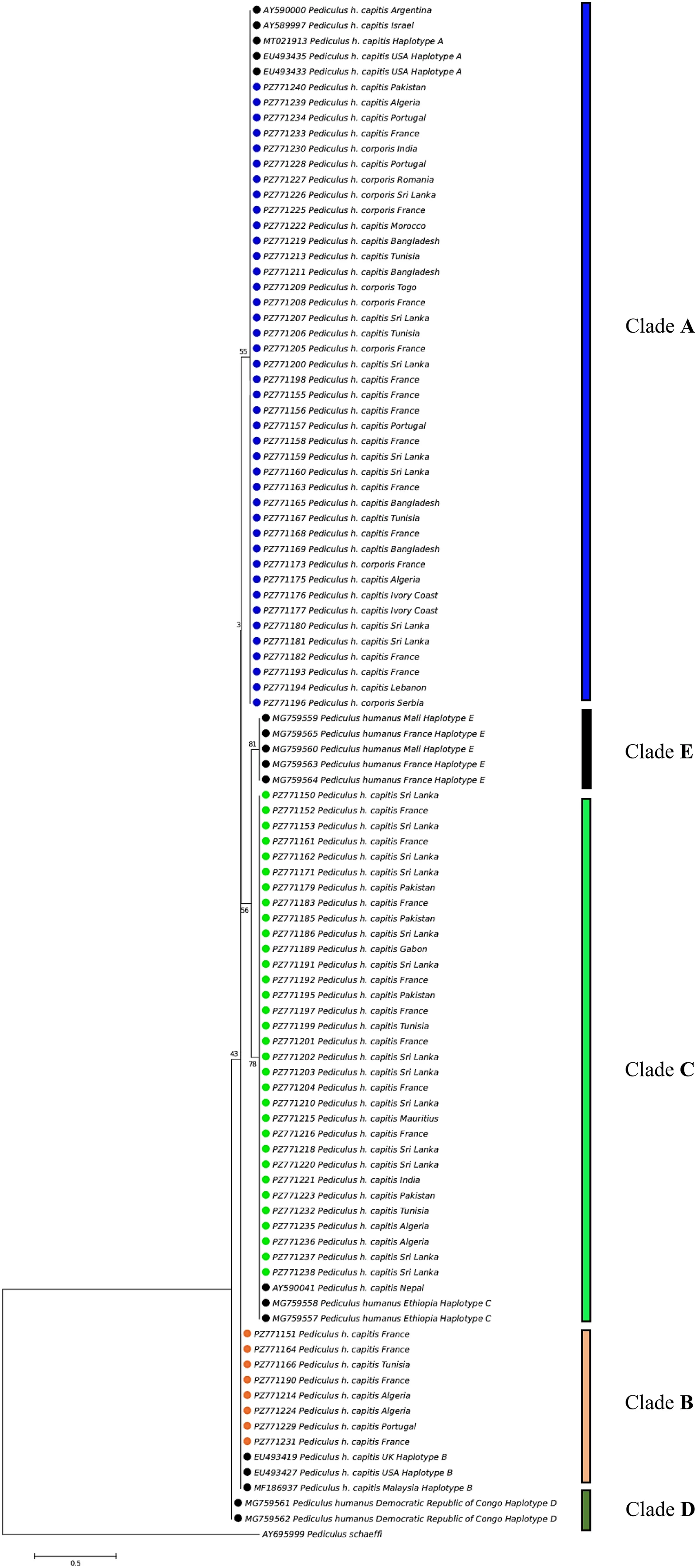
Maximum-likelihood phylogenetic analysis of mitochondrial cytochrome c oxidase subunit I (*COI*) gene sequences from head and body lice obtained in this study (color-coded and labelled with accession numbers) and reference sequences retrieved from GenBank (●).

**Figure 2:**
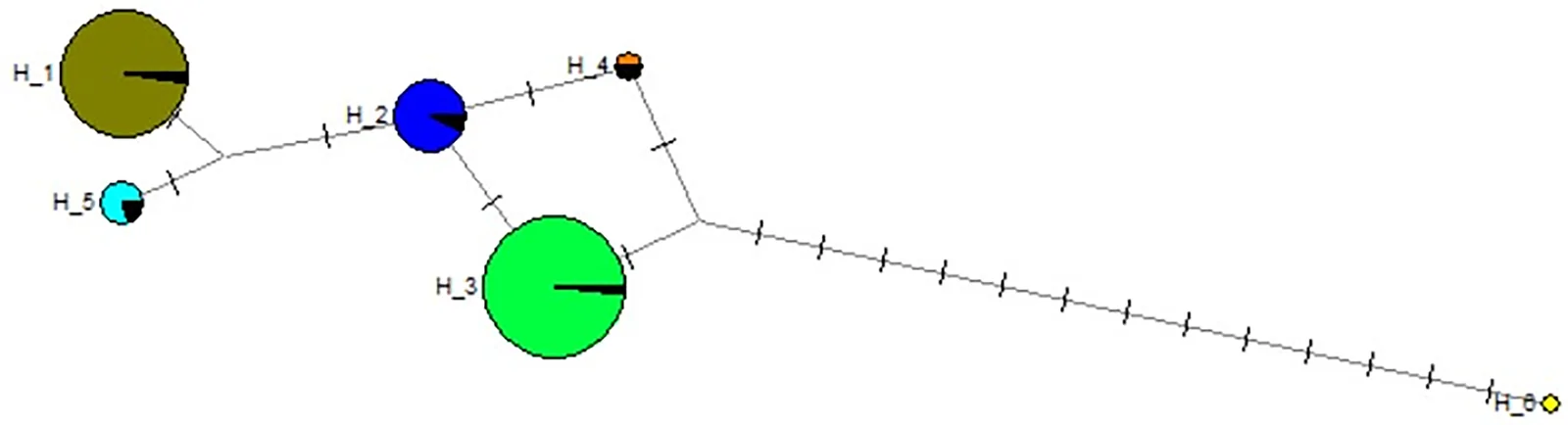
Global median-joining network analysis of head louse populations (haplotype distribution is provided in SI.IV). Haplotypes (H1–H3) comprised *Pediculus h. capitis* sequences, whereas H4 and H5 corresponded to *P. h. corporis*; *Pediculus schaeffi* (H6) was used as the outgroup.

## Discussion

Human lice infestations remain a common parasitic health concern in France, with several aspects of this parasitic infestation still poorly understood. Based on the available literature, about twenty studies addressing pediculosis have been published [6, 21, 29, 32]. Most of these studies focus primarily on head lice and their vectorial role, or treatment among school-aged children [19], with only a few studies examining broader clinical or molecular aspects [14, 15]. In the absence of comprehensive national prevalence data, local investigations have reported variable infestation rates, which also differ according to the lice species involved. Prevalence is reported to range from 3.3% for head lice among schoolchildren in Bobigny to 22% for body lice among homeless individuals in Marseille [12, 21]. However, knowledge regarding the epidemiological and clinical aspects of pediculosis across much of the country remains limited and fragmented, largely focusing to specific subpopulations – most notably school-aged children and homeless persons – leaving a substantial gap in knowledge regarding lice infestation in the general population and other age groups.

Avicenne Hospital is located in the northern suburbs of Paris, where many immigrants of diverse geographical origins reside, and where epidemiological information on pediculosis remains largely limited. In the prior study conducted by our Parasitology Department on patients presenting with lice infestations [14], a total of 259 lice were collected, including 235 head lice (from 141 patients) and 24 body lice (from 2 homeless patients). Female patients were the most frequently represented (male: female = 1:10). In the present study, conducted 8 years later in the same hospital in patients with pediculosis, a total of 209 specimens (one per patient) were analyzed, including head, body, and pubic lice, and examined using morphological and molecular approaches. Fewer than half of the patients originated from France (44.5%), while the remaining cases came from 17 countries across Africa (7 countries), Asia (5), Europe (4), and South America (1). The findings reported in this study together with our prior report [14] therefore represent the first investigations conducted in the general population with pediculosis, in contrast to previous studies that focused exclusively on schoolchildren [10, 21]. Furthermore, this study was the first to examine body and pubic lice in France. Although 75.8% of patients infested with head lice were schoolchildren, adults accounted for 24.2% of the cases. These results indicate that head lice infestation is not limited only to children and is also prevalent among adults. These findings are consistent with previous reports indicating adult infestation [35]. Additionally, head lice infestation was diagnosed in members of 71 families, highlighting the risk of infestation not only among affected individuals, but also among their family members. In contrast, body and pubic lice were most prevalent among adults, with age ranges from 35 to 84 years for body lice and from 32 to 73 years for pubic lice. Men were most frequently infested with both body (9/11 cases) and pubic lice (10/12 cases) than women. We also observed two infant cases, presumably infected by their parents, highlighting the risk of pubic lice infestation to close family members. It is noteworthy that four of the twelve patients with pubic lice were identified as MSM. To the best of our knowledge, this represents the first documented case of MSM with pubic louse infestation in France and the third reported worldwide [24, 43].

*Pediculus h. capitis* has been demonstrated to exhibit substantial genetic diversity with a phylogeographic classification comprising 6 clades, designated A to F. Head lice belonging to clades A and B have a worldwide distribution including countries on all continents, with clade A being reported more frequently [7, 31, 39]. In contrast, head lice belonging to clades C and D have been reported predominantly from Africa, followed by Asian countries [3, 33, 34]. Clade E appears mainly restricted to West Africa while clade F, the sister group of clade B, is mostly reported in South America [4, 9, 25]. In France, molecular investigations on the genetic diversity of human lice remain largely limited. The initial phylogenetic study in France, conducted by our department [14], revealed the presence of mitochondrial clades A, B, and E, the latter including *P. h. capitis* specimens isolated from immigrants from West Africa. Phylogenetic analysis of mitochondrial *COI* gene sequences in this study and those from GenBank revealed that the *P. h. capitis* specimens in the present study clustered into clades A, B, and C. The worldwide distribution of head lice belonging to clades A and B has been attributed to several factors, most importantly international travel and immigration [31, 37]. Despite the absence of clade E in this study, which was detected in our previous study [14], we report for the first time the presence of clade C in France, likely associated with the fact that 34% of the examined specimens originated from patients of Asian origin. Additionally, this is the first report documenting head lice belonging to clades A and C in countries like Sri Lanka, Bangladesh, and Pakistan (Fig. 1). Finally, based on BLAST and phylogenetic analysis, no genetic hybrids in *COI* sequences of head lice belonging to various clades were observed.

In addition, we were unable to trace the exact source of lice infestation in the patients examined in this study. The only confirmed information pertains to the origin of the infested patients themselves, not the origin of their lice infestation or their arrival time in France. However, based on the patients’ self-reported statement, most of them reported having been infested in their home countries. Furthermore, despite the limited number of body lice specimens analyzed in this study (11 specimens), phylogenetic analysis demonstrated that all belonged to clade A. This is therefore the first investigation examining the phylogenetic relationships of body louse specimens in France, and one of only a few in Europe.

## Conclusions

This study represents updated information on the epidemiological characteristics and genetic diversity of human lice infesting patients in the northern suburbs of Paris. Epidemiologically, head lice infestation was confirmed to be predominantly associated with children and their parents, particularly females, while body and pubic lice were mainly observed in adult males. The observed epidemiological patterns also emphasize that pediculosis affects diverse population groups with occasional intrafamilial (2 cases involving infants) or sexual (4 MSM cases) transmission, representing the first report of pediculosis among MSM in France. The identification of substantial genetic diversity among head lice, which clustered into 3 clades (A, B, C), including the first detection of mitochondrial clade C in France, together with the clustering of body lice within clade A, contributes to a better understanding of the genetic structure and evolutionary relationships of human lice. Although this study has some limitations, such as the inclusion of patients from a single hospital, the selective processing of samples (one specimen per patient), and the inability to determine the precise origin of infestation in some cases, these findings presented here significantly enhance our understanding of the genetic diversity of human lice and provide further insights into their evolutionary history. Future multicenter and longitudinal studies involving larger numbers of patients and lice from different regions of France are warranted to better characterize the national distribution and population structure of human lice. Broader geographic sampling and higher-resolution genomic approaches would further clarify transmission pathways, population connectivity, and the evolutionary history of circulating lice lineages. Such investigations could also help determine whether the emergence of clade C in France reflects a recent introduction, previously undetected circulation, or broader changes in the distribution and population structure of human lice.

## Data Availability

The datasets generated during the current study are available in GenBank, under accession numbers PZ771150–PZ771240.

## Supplementary material

*Supplementary File SI.I*: Epidemiological characteristics of patients with pediculosis. *Supplementary File SI.II*: Age range of patients with pediculosis. *Supplementary File SI.III*: *Pediculus humanus humanus* sequences deposited in GenBank. *Supplementary File SI.IV*: Haplotype distribution within processed louse sequences. The supplementary material of this article is available at https://www.parasite-journal.org/10.1051/parasite/2026060/olm.
