# Supplementary material for: Epidemiological and molecular insights into human lice (Anoplura: Pediculidae) in France: genetic diversity and DNA barcoding
Source: Parasite. 2026 Oct 13;33:60. doi: 10.1051/parasite/2026060 (PMC13661125; doi:10.1051/parasite/2026060)
Supplement: Supplementary file 1 [file parasite-33-60-s1.pdf]

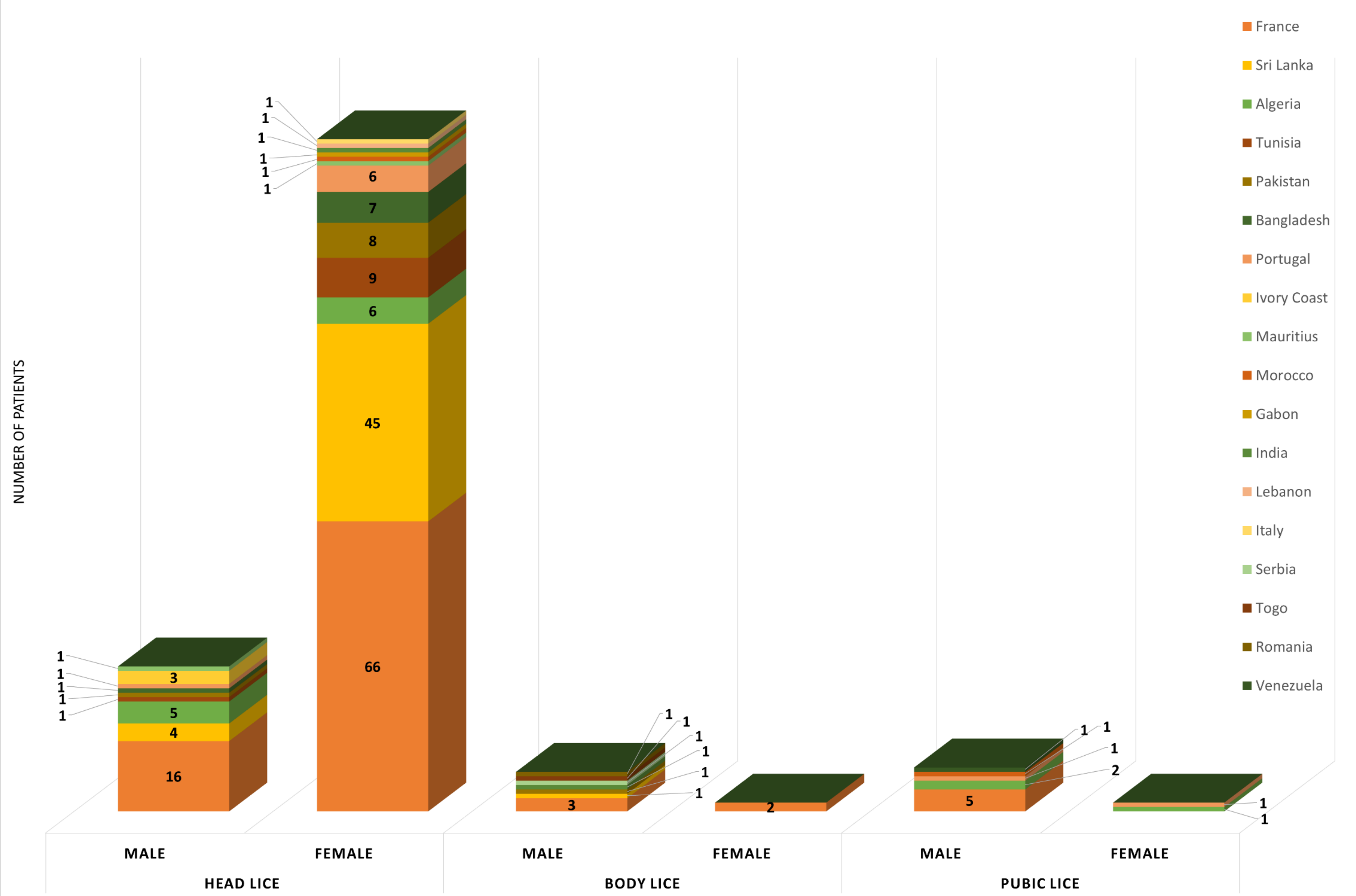

Supplementary file SI.I. Epidemiological features of the patients with pediculosis examined in this study.
